# Anemia and associated factors among children aged 6–23 months in Damot Sore District, Wolaita Zone, South Ethiopia

**DOI:** 10.1186/s12878-018-0108-1

**Published:** 2018-07-03

**Authors:** Bereket Geze Malako, Melese Sinaga Teshome, Tefera Belachew

**Affiliations:** 1 Damot Sore District Health Office, Wolaita zone, SNNPR, Gununo, Ethiopia; 2 Institute of Health, Public Health Faculty, Population and Family Health Department, Nutrition Course Team, Jimma University, Jimma, Ethiopia

**Keywords:** Anemia, Children, Damot sore, Wolaita

## Abstract

**Background:**

Anemia affects a significant part of the population in nearly every country in the globe. Iron requirements are greatest at ages 6–23 months when growth is extremely rapid and critically essential in critical times of life. Even though infants and toddlers are highly at risk, they are not considered as separate populations in the estimation of anemia. Despite this, the prevalence of anemia among under 24 months of age is still at its highest point of severity to be a public health problem in Ethiopia. There is no study that documented the magnitude of the problem and associated factors in the study area. The main aim of this study was to assess the prevalence of anemia and to identify associated factors among children 6–23 months of age.

**Methods:**

A community-based cross-sectional study was carried out among 485 children of Damot Sore, South Ethiopia from March to April 2017. Data on socio-demographic, dietary, blood samples for hemoglobin level and malaria infection were collected. Both descriptive and bivariate analyses were done and all variables having a *p*-value of 0.25 were selected for multivariable analyses. A multivariable logistic regression model was used to isolate independent predictors of anemia at a p-value less than 0.05. A principal component analysis was used to generate household wealth score, dietary diversity score.

**Results:**

Out of 522 sampled children, complete data were captured from 485 giving a response rate of 92.91%. For altitude and persons smoking in the house adjusted prevalence of anemia was 255(52.6%). The larger proportion, 128(26.4%) of children had moderate anemia. On multivariable logistic regression analyses, household food insecurity (AOR = 2.74(95% CI: 1.62–4.65)), poor dietary diversity (AOR = 2.86(95% CI: 1.73–4.7)), early or late initiation of complementary feeding (AOR = 2.0(95% CI: 1.23–3.60)), poor breastfeeding practice (AOR = 2.6(95% CI: 1.41–4.62)), and poor utilization of folic acid by mothers (AOR = 2.75(95% CI: 1.42–5.36)) were significantly associated with anemia.

**Conclusion:**

Prevalence of anemia among children (6–23 months) was a severe public health problem in the study area. Most important predictors are suboptimal child feeding practices, household food insecurity, and poor diet. Multi-sectoral efforts are needed to improve health and interventions targeting nutrition security are recommended.

## Background

Anemia is one of micronutrient deficiency which has serious and common public health significance in the world and it is the second leading nutritional cause of disability. Globally, about 42.6% of children (5–59 months) are suffering from anemia [1, 2]. It affects quarter of world population, primarily pregnant women and young children are at greatest risk [1].

There are many causes of anemia, of the iron deficiency (inadequate iron intake, poor iron absorption or excess iron losses), insufficient hematopoiesis (e.g., from vitamin B-12 deficiency), loss of blood (hemorrhagic anemia), premature red blood cell plasma membrane rapture (hemolytic anemia), deficient or abnormal synthesis of hemoglobin (e.g., thalassemia) or destruction of bone marrow (aplastic anemia were known [3, 4].

Children who live in Asia and Africa are at greatest risk with almost two-thirds of children living in Africa being anemic. Anemia can occur at any time and all stages of life cycle, but young children and pregnant women are the most at risk segment of the community. Iron requirements are greatest at ages 6–11 months when growth is extremely rapid [1, 2, 5].

Even if the prevalence of anemia among under-five children has dropped from 54% in 2005 to 40% in 2011, it increased significantly in Ethiopia according to EDHS 2016 report [6, 7]. Although there have been interventions by the government, prevalence of anemia is still at the highest point of severity to be a public health problem in Ethiopia. According to 2016 EDHS, the prevalence of anemia in this age category is 72.3% [7]. Regardless of efforts done so far, anemia remains to be public health problem especially in this age category [8, 9]. So, identifying factors associated is needed to develop appropriate interventions.

Thus, this study determined the prevalence of anemia among children of age 6–23 months and associated factors in Damot Sore District, Wolaita Zone, South Ethiopia.

## Methods

### Study area and sample

This study was conducted in Damot Sore District located 326kms south of Addis Ababa. According to the district health office, the total population of the study area for the year 2016/2017 was 128,184 and total numbers of children (6–23 months) were 4499. The altitude of the district is between the ranges of 1500-2500 m. A community-based cross-sectional study was conducted from March 10 to April 10, 2017.

All children aged 6–23 months that were residents of the district for more than 6 months were included, on the other hand, children who had received de-warming, received blood transfusion and diagnosed of anemia and on medication prior (2 months) to data collection and child or mother with health condition that hinders verbal communication were excluded from study. The sample size was estimated using with Epi InfoTM7 by using formula for estimation of a single population proportion for anemia and two population proportion formulas for associated factors with the following assumptions: prevalence of anemia among children 6–23 months of 66.6, 95% confidence level and 5% margin of error from prior study [8] and multiplying 1.5 for the design effect and adding a non-response rate of 10%. Therefore, the subsequent reports were based on the total sample of 522.

Multistage sampling was used to select study participants. For this, first, 6 Kebeles (smallest political administrative unit of government) were selected using lottery method out of 20 “Kebles” in the District. Secondly, within those Kebeles, simple random sampling was used to select the study participants using Kebele health posts family folders of community health information system (CHIS). Households with children 6–23 months were selected using a family folder as a sampling frame.

### Measurements

Socio-demographic and economic data were collected by interviewing mothers of the children during house to house data collection by using pretested and interviewer administered a questionnaire that was prepared in the English language, which later was translated into Wolayttattua-local language. The standardized tool for measurement of wealth index was adapted from EDHS 2011 [6]. To determine dietary practices of children food frequency questionnaire [(FFQ) were used. The frequency of consumption of food items by children during the last 1 month prior to data collection was asked. FFQ was developed after pilot study on 5% of a sample size of 24-h recall conducted to list out all foods which were be eaten as a complementary food, and then key informants were asked for additional listing and prioritization, the approach was used in earlier studies [10].

Altitudes of Kebeles were measured by using a hand-held Global Positioning System; Mark: GPS 72H GARMIN. Hemoglobin levels were adjusted for altitude changes. Similarly, hemoglobin levels were adjusted for children who live with smoking individual within the household that smokes indoor [11].

Laboratory investigations were done for hemoglobin measurement and malaria status. Hemoglobin measurement was measured from capillary blood by collecting one drop of blood carefully from the middle finger. The finger of the child pricked after rubbing the fingertip with sterile cotton (immersing in 70% alcohol) with a sterile disposable lancet. Automated HEMOCUE Hb 301, HEMOCUE AB, ANGELHOLM SWEDEN machines were used to determine the hemoglobin concentration, which was recommended elsewhere for the survey in resource-poor settings and the results were expressed in g/dl, then categorized based on criteria of WHO cut off point [11, 12].

Malaria test was also done using rapid diagnostic test (RDT), which was appropriate for community survey to know malaria status [13].

### Data analysis

Data were entered into EPI data version 3.1 and then exported to SPSS for data analysis. Statistical analyses were performed using a computer software package SPSS for Windows, version 21.0 (SPSS, Chicago, IL, USA)). Frequency distributions of socio-demographical, environmental health and sanitation related variables, household food insecurity data were first explored using frequencies and cross-tabulations by anemia status and the then binary logistic regression model was fitted with anemia as a dependent variable.

Bivariate analysis was done and All variables which had the association with the outcome variable at *p* < 0.25 on bivariate analyses were selected for entry into multivariable logistic analyses, which was used to isolate independent predictors of anemia by adjusting for other variables. Adjusted odds ratio (AOR) with 95% Confidence interval (CI) were used to determine the strength of association. Variables with *P* < 0.05 were considered to have statistical significance.

Principal Component Analyses (PCA) were done for household wealth score and dietary diversity score. Then ranked into tertiles (low, middle and high) for wealth and dietary diversity and then dichotomization was done for dietary diversity. The lowest two tertiles were leveled as poor DDS, the highest ranks were categorized as good DDS. Regarding dietary practices, a similar approach was used in a study conducted in Jimma, Ethiopia and elsewhere [14, 15].

An ethical approval was obtained from the Ethical Review Board of Jimma University. Written and informed consents were secured from each mother of children. Children who were found to have malaria were given free treatment from the health posts. Similarly, parents of children diagnosed with severe anemia were given referral papers go to the nearby health center.

### Data quality

A two-day training was given for data collectors regarding study objectives, interviewing techniques and ethical issues during data collection. A pretest was done among 5% of the total sample size in Shayamba Kilena Kebele which was not selected into the sample. The questionnaires were checked daily for accuracy, consistency, and completeness.

Proper functionality and technical performance of instruments were cross-checked by using quality control samples, for malaria test result with blood film result by microscope and for Hemocue with CBC machine were checked. Comparisons of Hemocue machines with CBC (Complete blood count) machine, Sysmex analyzer (Sysmex XS-500i, made in China) were done. This was just to be confident on the working instruments by themselves but not on technical issues behind the machines, that how they measure. The results of Pearson correlation coefficients for survey Hemocue machines with Sysmex analyzer were:0.979,0.987, 0.996 and 0.995.

Standard operating procedures and manufacturer’s instruction were strictly followed starting from sample collection up to result reporting for laboratory activities. All laboratory procedures were handled by laboratory technologists. Before data analyses cleaning were done and also out layers were identified and managed. Crombach’s alpha was checked for household wealth and food frequency.

## Results

Out of the total 522 children were included in this study, 485 responded giving a response rate of 92.91%. Twenty-seven 27(7.89%) of sampled children were not included in the analysis due to the incompleteness of their data (Table 1).

**Table 1:** Socio-demographic and economic and care-related characteristics of the family and children 6–23 months aged in Damot Sore District, Wolaita Zone, South Ethiopia, from March to April 2017

Characteristics	Categories	Frequency (*n* = 485)	Percent (%)
Sex of the children	Male	247	50.9
	Female	238	49.1
Age of mothers(years)	15–24	86	17.7
	25–34	267	55.1
	35–49	132	27.2
Age of the children (months)	6–11	193	39.8
	12–17	164	33.8
	18–23	128	26.4
Educational status of mothers	No formal education	312	64.3
	Formal education	173	35.7
Educational status of fathers	No formal education	243	50.1
	Formal education	239	49.3
Mothers occupation	Unemployed	467	96.3
	Government/private employee	18	3.7
Fathers occupation	Unemployed	462	95.3
	Government/private employee	19	3.9
Family size	Less than or equal to 5	221	45.6
	Greater than 5	264	54.4
Under five children within household	More than one child	201	41.4
	One child	284	58.6
Household Wealth	Low	204	42.1
	Middle	74	15.3
	High	207	42.7
Introduction time of complementary feeding	Before or after 6 months	226	46.6
	At 6 months	259	53.4
Breast feeding practice of mothers	Poor	115	23.7
	Good	336	69.3
	Not breast feed	34	7.0
Appropriately utilized iron folate	For less than or equal to 3 months	331	68.2
	For greater than 3 months	62	12.8
	Not utilized at all	92	19
Source of drinking water	Piped inside compound	37	7.6
	Public	357	73.6
	Protected well/spring	91	18.8
Toilet	No facility/bush/field	12	2.5
	Local pit latrine	468	96.5
	VIP latrine	5	1.0
Utilization of ITN	No	46	9.5
	Yes	411	84.7
Diarrhea	No	75	31.2
	Yes	165	68.8
Malaria	No	464	95.7
	*P. falciparum*	6	1.2
	P.vivax	8	1.6
	P.mixed	7	1.4
Surgery	No	481	99.2
	Yes	4	0.8
DDS	No	319	65.8
	Yes	166	34.2
Fermented foods preparation	No	299	61.6
	Yes	186	38.4
Germinated or soaked foods	No	379	78.1
	Yes	106	21.9

Out of 485 children, 247(50.9%) were males and 238(49.1%) were females. The age range of children was 6–23 months and the mean ages were 13.65 ± 5.401 months and while their mother’s age range was 15–45 years and their mean age was 30.12 ± 5.807 years. Regarding the severity of anemia, among sampled children, 106(21.9%) were mildly anemic, 128(26.4%) were moderately and 21(4.3%) were severely anemic (Table 1).

In Bivariate analysis a total of 15 variables with a *p*-value < 0.25 were entered into multivariable logistic regression and finally 5 variables (household food insecurity, dietary diversity, introduction time of complementary feeding, iron folate utilization of mothers and quality of breastfeeding) with p-value < 0.05 were isolated to show association with anemia independently. Children with poor dietary diversity score were nearly three times more likely to be anemic than children with good dietary diversity scores (AOR = 2.86 (95% CI: (1.73–4.70)) (Table 2).

**Table 2:** Independent predictors of anemia among children 6–23 months aged in Damot Sore District, Wolaita Zone, South Ethiopia, from March to April 2017

Predictors	Anemia status		COR (95% C.I.)	AOR (95% C.I.)	p
	Anemic	Not anemic			
DDS			2.89(1.96–4.28)		0.01
Poor	196(40.4%)	123(25.4%)		2.86(1.73–4.70)	
Good	166(34.2%)	107(22.1%)		1.00	
Introduction time of complementary feeding	142(29.4%)	82(17%)	2.3(1.60–3.33)	2.0(1.23–3.60)	0.01^a^
Before or after 6 months of age					
At 6 months of age					
	111(23%)	148(30.6%)	1.00	1.00	
IFA utilization history	177(45%)	154(39.2%)	2.2(1.27–3.96)	2.75(1.42–5.36)	0.03
≤ 3 months	21(5.3%)	41(10.4%)	1.00	1.00	
> 3 months					
Breast feeding practice	82(18.2%)	33(7.3%)	2.97(1.90–4.70)	2.6(1.41–4.62)	0.02
Poor	153(33.9%)	183(40.6%)	1.00	1.00	
Good					
Household food insecurity	212(43.7%)	119(24.5%)	4.6(3.0–6.90)	2.74(1.62–4.65)	0.04
Food insecure	43(8.9%)	111(22.9%)	1.00	1.00	
Food secure					

^a^Variable with *p*-value< 0.001

Children those introduced complementary feeding earlier than the recommended time, which is at 6 months, or those started late after recommended time were nearly 2 times more likely to be anemic than children which started complementary feeding at 6 months (AOR = 2.0(95% CI: 1.23–3.60) (Table 2).

Children living in food-insecure households were 2.7 times more likely to be anemic than food secure households (AOR = 2.74(95% CI: 1.62–4.65)). Children of mothers those utilized iron folate for less than or equal to 3 months (90 days) during pregnancy were 2.75 times more likely to be anemic than mothers of those utilized more than 3 months children (AOR = 2.75(95%CI:1.42–5.36)). Children from mothers who have had a poor practice of breastfeeding were three times more anemic than children of mothers that have had good breastfeeding practice (AOR = 2.6(95% CI: 1.41–4.62)) (Table 2).

## Discussion

This study showed that overall prevalence of anemia among children 6–23 months was 52.6%. After adjusting for various socioeconomic and demographic variables, care related, environmental health and sanitation, dietary and household food modifications, household, and morbidity related variables: having poor dietary diversity, early or late initiation of complementary feeding, poor quality of breastfeeding and children of mothers less utilizes iron folate during pregnancy. The prevalence level of anemia observed in this study among children 6–23 months were classified by WHO as a severe public health problem [16].

Prevalence of anemia in this study was higher than the prevalence of EDHS 2016 regional report for SNNPR which was 49.6% [7]. The possible reasons for the variation may be due to differences in samples size, lifestyles and other reason might be socioeconomic status variation. This might be due to fasting time for Orthodox Christianity followers and due to seasonal food scarcity that reduces consumption of diversified foods, as the data were collected in spring “Belg”, which were the dry and sunny season and this leads to micronutrient deficiency-related diseases such as anemia.

But it was much lower than EDHS 2016 as a country whole which was 72.7% [7], and also a study conducted in Wag-Himra zone in north Ethiopia which was 66.6% [8]. From abroad still, it was much lower than studies done in Cameroon [17] and Sudan [18] with the prevalence of 66.7 and 86%, respectively. The lower prevalence in this study might be due to the change made by the existing nutritional, public health interventions, easy accessibility of health information through health extension workers and among other factors may be included. But still it lacks adequacy of the sample size to compare with the national data of EDHS with this small cross-sectional study, thus it needs large sample size and also analytic studies to know temporal and seasonal variations.

Regarding food insecurity, children living in food-insecure households were more likely to be anemic than food secure households. This result disagrees with the findings reported from Iran [19] and India [20] that showed, as there were no relations with household food insecurity and anemia status of children. However, this finding was in-line with the study findings from USA [21]. In our study, association with household food insecurity might be due to climate change (El-Niño effect) which shifted seasonal rainfall, reduced yield, and agricultural productivity. So, to cope with food insecurity at the household level, children as other household members reduce consumption of diversified foods (especially, animal source iron-rich and enhancing or vitamin C rich foods) worsen childhood anemia.

Children with poor dietary diversity score were more likely to be anemic than children with good dietary diversity scores. Studies regarding dietary practices of children in some parts of Ethiopia showed that grains, roots, and tubers were eaten by 80.2% of children. And these foods are a source of non-heme iron with low bioavailability and with higher content of phytates, phenols and make children prone to nutritional related deficiencies [22]. This in-line with a study conducted in northern Ethiopia that showed, those children who consumed cereal based monotonous diet were 3.2 times more anemic than those consume diversified diet [8]. In our study, this might be due to tuber, root and cereal-based monotonous diet. In addition, cultural and economic reasons do not allow children to eat iron-rich foods such as meat. In addition to this seasonal unavailability of enhancing citrus fruits such as lemons and oranges might be among reasons. Thus, monotonous diet consumption of cereals and tubers, reduced consumption of iron-rich foods, enhancing [citric fruits] and inhibitors [coffee and tea] were made children be anemic [23].

Children who started complementary feeding earlier than or late to recommended time by WHO, which was at 6 months, were two times more likely to be anemic than children which started complementary feeding just at 6 months. Contrary to this, a study conducted in Nepal on the age of introduction for complementary feeding for infants revealed that early introduction of solid foods at three and 4 months respectively has a significant improvement of iron status among children in developing countries [24]. However, our findings in-line with studies done in Wag-Himra, Ethiopia [8], Brazil [25] and China [26] which revealed that a lower infant Hb was associated with early initiation of complementary feeding and increased EBF duration for more than 6 months. Early exposure of infants (before 6 months of age) to microbial pathogens due to complementary foods increases the risks of infection for diarrheal diseases, thereby leading to mal-absorption (environmental enteropathy). Breast milk has minimal iron to fulfill nutritional requirements of a growing infant, given that providing breast milk alone coupled with rapid iron depletion beyond 6 months also increases the risk of anemia for younger infants [27]. In our study, this might be due to cultural belief of mothers that exclusive breastfeeding is inadequate to infants alone, and they introduce cow milk earlier than 6 months.

Children of mothers those utilized iron folate for less than 3 months during pregnancy were more likely to be anemic than those utilized IFA for more than 3 months. This was similar to a study conducted in India, as children hemoglobin levels were independently associated with maternal hemoglobin level, and antenatal anemia contributes to low birth weight and prematurity, both of which increase the risk of childhood anemia. Severe maternal anemia may also reduce breast milk iron content [20]. In this study, it might due to late initiation of antenatal care, timely shortage of iron folate in health facilities, fear of side effects and lack of awareness among mothers about iron folate had made mothers not to utilize as recommended by the national guideline. These factors might have made a lessened store of iron in mothers to transfer an adequate amount of iron for the fetus. A little store of iron in children during delivery would be a risk factor for anemia in their infancy [5].

Our study showed that children from mothers who had a poor practice of breastfeeding had a statistically significant association with anemia. This study is in-line with other studies conducted in Iran [28], China [26] and Brazil [29] that reported breastfeeding for less than 6 months or exclusive breastfeeding for more than 6 months, early initiation of solid or liquid foods, appropriate position of attachment, switching time from one breast to another and low frequency of breastfeeding per day were associated with childhood anemia. Similarly, in our study, this might be due to lack of awareness among mothers about good breastfeeding practices and lack of advice from health care providers. Mothers were busy with domestic works and greater caring responsibility of the whole family and domestic animals [29].

Although Ethiopia has tried many actions such as NNP, CBN, social protections and others, to reduce nutritional deficiencies, the prevalence of anemia among children remained high over the last few years [6–8]. A landscape analysis and a study conducted in Ghana revealed that there was a significant decline in overall anemia prevalence in children age 6–59 months between 2008 and 2014 (from 78 to 66%). This happened due to multi-sectoral collaboration, daily iron, and folic acid supplementation in pregnant women, intermittent iron and folic acid supplementation in menstruating women and home fortification of foods with multiple micronutrient powders among infants and children 6–23 months, and wheat and maize flour fortification with sodium iron ethylene ediaminetetraacetic acid with malaria prevention [30, 31].

Evidence on the impact of scaled-up iron supplementation across countries in the world on the reduction of anemia prevalence documented that, national programs of Thailand and Nicaragua decreased the prevalence of anemia among children through improving iron folate utilization by pregnant mothers. As they reported in their document, they improved [reduced) the prevalence of anemia by strengthening already existing policy, adopting lessons from successful countries and by strengthening demand and supply system [32].

Food security and diversified diet were important for proper child growth and remains a concern in Ethiopia. There are Programs that would be awaited for changing nutritional problems to scale up, like Seqota declaration [33, 34], and some social protections were implementing. Productive Safety Net Program (PSNP) as one of social protection for food insecure households, were to improve household food security but evaluations of the first three phases of PSNP demonstrated a two-month improvement in food security, but lack of improvement in the quality of children’s (6–23 months) diets [33].

Solutions, such as nutrition-sensitive agriculture-specific activities: agriculture based solutions, such as production of nutrient dense crops, livestock/livestock products, agro-processing/storage skills, increasing on-farm or off-farm income for vulnerable households and women need to be further established in order to increase the production and access to diverse, safe, and nutrient-dense foods were considered by government of Ethiopia [33, 34].

It was well documented in a systematic review of thousand day’s interventions, interventions in critical times improve irreversible cognitive impairment due to reduced iron. It is a window of opportunity with minimal cost that adds national economy, creative minds and makes healthy old age [5].

Although the micronutrient prevention and control guideline has been developed since 2005 and there have various intervention to prevent anemia at the community level, our results showed the existence high prevalence among children. The practical implication of this updated level of prevalence is, even though it is an area specific, it will use as an input for national nutritional program goal and for Growth and Transformation Plan GTP 2 (2016–2020), which were targeted to reduce micronutrient deficiencies and other nutritional problems. In addition to this, it is an input for declarations that programmed to end up malnutrition problems which were planned by the government such as Seqota declarations. As a country government of Ethiopia planned to be the middle-income country in the year 2030. Sour findings will help FMoH as an input for fortifying its efforts to achieve its plan of enhancing the productivity of individuals who will contribute to the achievement of sustainable development goals.

### Limitations of the study

We acknowledge a number of limitations in our study. A cross-sectional design enabled us only to assess hemoglobin levels at one point in time. It is documented in other studies that as intestinal parasites, hereditary diseases, chronic illnesses such as HIV and others could cause anemia, which was not assessed in this study. There were some recall biases among mothers of younger children regarding introduction time of complementary feeding. We minimized this by asking them to remember a time when at least they introduced to water or cow’s milk.

## Conclusion and recommendations

The prevalence of anemia among children 6–23 months in Damot Sore District, South Ethiopia was 52.6% indicative of the fact that anemia is high public health important problem by WHO classification criteria. Initiation time of complementary feeding, breastfeeding practice, maternal history of iron-folate (IFA) utilization, dietary diversity and household food insecurity were significantly associated with anemia.

Because of this severity of anemia, it requires public health intervention and by taking this into account, the following recommendations are forwarded: Strengthening community-based nutrition activities which were previously integrated into health extension packages. Providing community-based nutrition education on the feeding of diversified diets, increase vitamin C rich foods, reduced consumption of foods that prevent iron absorption (E.g. coffee and tea) and to take an interval to take a milk after consumption of iron-rich foods. Encourage mothers for early follow-up of ANC, IFA utilization, good breastfeeding practices and the right initiation time of complementary feeding. Strengthening integration of services within health facilities. Strengthening activities on identifying households, those in food insecure status and include them in social protection packages. Further analytic studies for temporal relationships of above factors.

## Abbreviations

ANC: Antenatal care
AOR: Adjusted odds ratio
CBC: Complete blood count
CBN: Community-based nutrition
CHIS: Community health information system
CI: Confidence Interval
COR: Crude odds ratio
DDS: Dietary diversity score
EBF: Exclusive breast feeding
EDHS: Ethiopian demographic and health survey
FFQ: Food frequency questionnaire
GPS: Global positioning system
Hb: Hemoglobin
HFIAS: Household food insecurity access scale
IFA: Iron folic acid
ITN: Insecticide treated bed net
NNP: National nutrition programme
OR: Odds ratio
PCA: Principal component analysis
RDT: Rapid diagnostic test
SNNPR: South nation’s nationalities and peoples regional state
VIP: Ventilation improved pit latrine
WHO: World Health Organization

## References

[1] McLean E, Cogswell M, Egli I, Wojdyla D, Benoist B (2008). Worldwide prevalence of anaemia, WHO vitamin and mineral nutrition information system: 1993-2005. Public Health Nutrition.

[2] WHO. The global prevalence of anaemia in 2011. Geneva, Switzerland 2015 [Accessed: 02 Feb 2017]; Available from: http://apps.who.int/iris/bitstream/10665/177094/1/9789241564960_eng.pdf.

[3] Ganong WF. Review of Medical Physiology, Twenty-Second Edition University of California, San Francisco, USA: McGraw-Hill Companies; 2003.

[4] Guyton AC, Hall JE. Text Book of Medical Physiology, thirteenth edition. Jackson, Mississippi, USA: Elsevier Inc.; 2006; 11.

[5] Burke RM, Leon JS, Suchdev PS (2014). Identification, prevention and treatment of Iron deficiency during the first 1000 days. Nutrients.

[6] C.S.A. Ethiopia Demographic and Health Survey 2011. Addis Ababa, Ethiopia 2012 [8 Feb 2017]; Available from: https://www.unicef.org/ethiopia/ET_2011_EDHS.pdf.

[7] C.S.A. Ethiopia Mini Demographic and Health Survey 2016 Key Indicators Report. Addis Ababa, Ethiopia 2016 [Accessed: 01 Feb 2017]; Available from: https://dhsprogram.com/pubs/pdf/PR81/PR81.pdf.

[8] Woldie H, Kebede Y, Tariku A (2015). Factors Associated with Anemia among Children Aged 6–23 Months Attending Growth Monitoring at Tsitsika Health Center, Wag-Himra Zone, Northeast Ethiopia. Journal of Nutrition and Metabolism.

[9] Roba KT, O’Connor TP, Belachew T, O’Brien NM. Concurrent iron and zinc deficiencies in lactating mothers and their children 6–23 months of age in two agro-ecological zones of rural Ethiopia. Eur J Nutr. 2018;57(2):655-67. 10.1007/s00394-016-1351-5 10.1007/s00394-016-1351-5 27942846

[10] Fatihah F, Ng BK, Hazwanie H, Norimah K, SiN S, Ruzita AT (2015). Development and validation of a food frequency questionnaire for dietary intake assessment among questionnaire for dietary intake assessment among multi-ethnic primary school-aged children Singapore. Medical Journal.

[11] WHO. Haemoglobin concentrations for the diagnosis of anemia and assessment of severity. In: System. VaMNI, editor. Geneva, World Health Organization 2011 (WHO/NMH/NHD/MNM/11.1).

[12] Nkrumah B, Nguah SB, Sarpong N, Dekke D, Idriss A, May J (2011). Hemoglobin estimation by the HemoCue® portable hemoglobin photometer in a resource poor setting. BMC Clin Phatol.

[13] Unicef. Malaria diagnosis: a guide for selecting rapid diagnostic test (RDT) kits - 1st edition Copenhagen 2007 [Accessed 17 Jan 2016]; Available from: https://www.unicef.org/french/supply/files/Guidance_for_malaria_rapid_tests.pdf

[14] Belachew T, Lindstrom D, Gebremariam A, Hogan D, Lachat C, Huybregts L (2013). Food Insecurity, Food Based Coping Strategies and Suboptimal Dietary Practices of Adolescents in Jimma Zone Southwest Ethiopia. PLoS One.

[15] Thorpe MG, Milte CM, Crawford D, McNaughton SA (2016). A comparison of the dietary patterns derived by principal component analysis and cluster analysis in older Australians. International Journal of Behavioral Nutrition and Physical Activity.

[16] WHO/UNICEF/UNU. Iron deficiency anaemia: assessment, prevention, and control. Geneva: World Health Organization; 2001. (WHO/NHD/01.3).

[17] Sop MMK, Mananga M-J, Tetanye E, Gouado I (2015). Risk factors of anemia among young children in rural Cameroon. Int J Curr Microbiol App Sci.

[18] Mahmoud HH, Muddathir AM, Osman SEM, MA AK (2014). K A. Iron Deficiency Anemia among Children under Three years in Kassala, Eastern Sudan. Sudanese J Public Health.

[19] Salarkia N, Neyestani TR, Omidvar N, Zayeri F (2015). Household Food Insecurity, Mother’s Feeding Practices, and the Early Childhood’s Iron Status. Int J Prevent Med.

[20] Pasricha S-R, Black J, Muthayya S, Shet A, Bhat V, Nagaraj S (2010). Determinants of Anemia among young children in rural India. Pediatrics.

[21] Park K, Kersey M, Geppert J, Story M, Cutts D, Himes JH (2009). Household food insecurity is a risk factor for iron-deficiency anemia in a multi-ethnic, low-income sample of infants and toddlers. Public Health Nutrition.

[22] Beyene M, Worku AG, Wassie MM (2015). Dietary diversity, meal frequency and associated factors among infant and young children in Northwest Ethiopia: a cross- sectional study. BMC Public Health.

[23] Michaelsen KF, Hoppe C, Roos N, Kaestel P, Stougaard M, Lauritzen L (2009). Choice of foods and ingredients for moderately malnourished children 6 months to 5 years of age. Food Nutrition Bulletin.

[24] Chandyo R, Henjum S, Ulak M, Lyman AT, Ulvik R, Shrestha P (2016). The prevalence of anemia and iron deficiency is more common in breastfed infants than their mothers in Bhaktapur, Nepal. Eur J Clin Nutri.

[25] Jordão RE, Bernardi JLD, AdAB F (2009). Feeding pattern and anemia in infants in the city of Campinas, São Paulo, Brazil. Rev Paul Pediatr.

[26] Luo R, Shi Y, Zhou H, Yue A, Zhang L, Sylvia S (2014). Anemia and feeding practices among infants in rural Shaanxi Province in China. Nutrients.

[27] Iannotti LL, Tielsch JM, Black MM, Black RE (2006). Iron supplementation in early childhood: health benefits and risks. Am J Clin Nutr.

[28] Dalili H, Baghersalimi A, Dalili S, Pakdaman F, Rad F, Kakroodi A (2015). Is there any relation between duration of breastfeeding and anemia?. Iranian J Pediatr Hematol Oncol.

[29] Castro T, Baraldi L, Muniz P, Cardoso M (2009). Dietary practices and nutritional status of 0–24-month-old children from Brazilian Amazonia. Public Health Nutrition.

[30] Adu-Afarwuah S, Lartey A, Brown KH, Zlotkin S, Briend A, Dewey KG (2008). Home fortification of complementary foods with micronutrient supplements is well accepted and has positive effects on infant iron status in Ghana. Am J Clin Nutr.

[31] SPRING GHS. Ghana Landscape Analysis of Anemia and Anemia Programming In: Strengthening Partnerships R, and Innovations in Nutrition Globally (SPRING) project., editor. Arlington, VA 2016.

[32] Sanghvi TG, Harvey PWJ, Wainwright E (2010). Maternal iron–folic acid supplementation programs: evidence of impact and implementation. Food and Nutrition Bulletin.

[33] FMoH/UNICEF/EU. Situation Analysis of the Nutrition Sector in Ethiopia: 2000-2015. Addis Ababa, Ethiopia 2016; Available from: https://www.unicef.org/ethiopia/ECO_Nutrition_Situation_Analysis_Main_Document.pdf.

[34] MoANR. The Federal Democratic Republic of Ethiopia Ministry of Agriculture and Natural resource Nutrition Sensitive agriculture: draft Strategic Plan. Addis Ababa, Ethiopia 2016 [Accessed: February, 2017]; Available from: http://www.moa.gov.et/documents/93665/6308679/Nutrition+Sensitive+agriculture+draft+Strategic+Plan.pdf/2b710b04-2cea-4442-b74c-3ea4033637ef.

